# A Multimodal Acousto-Optic Dataset for Underwater Image Enhancement, Detection, and Reconstruction

**DOI:** 10.1038/s41597-026-07116-3

**Published:** 2026-04-06

**Authors:** Xuanhe Chu, Shijian Zhou, Junwen Tian, Dehua Zou, Xinyu Zhao, Ruixue Wang, Bin Cheng, Yuhan Dong, Songhao Zhao, Han Chen, Zhiying Jiang, Jie An, Minyi Xu, Xianping Fu, Siyuan Liu

**Affiliations:** 1 Dalian Maritime University, Marine Engineering college, Dalian, 116026 China; 2 Dalian Maritime University, Marine Electrical Engineering college, Dalian, 116026 China; 3 Dalian Maritime University, Information Science and Technology college, Dalian, 116026 China; 4 Dalian Maritime University, State Key Laboratory of Maritime Technology and Safety, Dalian, 116026 China

**Keywords:** Ocean sciences, Scientific data, Computer science

## Abstract

With the development of acoustic and optical exploration technologies, devices such as optical camera, laser scanner and multibeam sonar provide the possibility of underwater accurate perception. The optimization of acoustic and optical data through algorithms have become one of the major concerns in underwater computer vision field. However, these optimization and improvement algorithms require a large amount of underwater multimodal data for training and evaluation. To address these needs, we propose a multimodal acousto-optic dataset for underwater image enhancement, detection, and reconstruction (MAOUD), which was collected from our underwater simulation environment and includes optical RGB images, acoustic images, labeled images, laser point clouds and acoustic videos. To ensure the accuracy of the dataset, we used a state-of-the-art underwater multimodal integrated detector with guaranteed corresponding kinematic parameters. The dataset can be used for training and evaluation work for a variety of underwater acoustic and optical tasks, serving as a standardized training and validation benchmark for multimodal underwater acoustic and optical algorithms, which holds significant importance for advancing underwater exploration technology.

## Background & Summary

Underwater environment detection technology^1^ is now one of the main concerns in the field of ocean exploration and underwater computer vision. Previously, there have been acoustic^2–4^ or optical^5–8^ public datasets for underwater target detection^9,10^ and 3D reconstruction^11,12^ and other underwater detection tasks. Tables 1 and 2 present the types of tasks supported by these existing single-modal datasets, while Table 3 provides a detailed quantitative comparison between our MAOUD and other underwater optical and acoustic datasets, covering key metrics such as total number of images, number of annotated images, number of object categories, image resolution, sensor modalities provided, and acquisition environments. However, with the development of underwater multimodal detection technology^13^, acoustic or optical datasets that only provide single-modal data are often unable to satisfy the research needs, and it becomes particularly important to provide underwater datasets with acoustic-optical multimodal data. The goal of this paper is to perform acoustic-optical detection, enhancement and reconstruction tasks simultaneously in an underwater simulated environment in the same region with a large number of training samples and multi-source data categories.

**Table 1:** The “*✓*” mark indicates the types of tasks that can be performed on optical datasets (enhancement, detection, reconstruction).

Optical Dataset	Enhancement	Detection	Reconstruction
Sea-Thru	*✓*		*✓*
MIMIR-UW			*✓*
UIEB	*✓*		
RUIE	*✓*	*✓*	
MAOUD	*✓*	*✓*	*✓*

**Table 2:** The “*✓*” mark indicates the types of tasks that can be performed on acoustic datasets (enhancement, detection, reconstruction).

Acoustic Dataset	Enhancement	Detection	Reconstruction
UATD		*✓*	
NKSID		*✓*	
SCTD		*✓*	
MAOUD	*✓*	*✓*	*✓*

**Table 3:** Quantitative comparison of MAOUD with existing underwater datasets, including total number of images, number of annotated images, number of object categories, image resolution, sensor modalities provided, and acquisition environment.

Dataset	Image	Annotation	Object category	Resolution	Sensor Modality	Environment
Sea-Thru	93	0	0	1776 × 1182	Optical Only	Real
UIEB	950	0	0	640 × 360	Optical Only	Real
RUIE	3,630	300	4	400 × 300	Optical Only	Real
MIMIR-UW	>10,000	0	0	720 × 540	Optical Only	Virtual
UATD	9,200	7,400	10	1024 × 1428	Acoustic Only	Real
NKSID	2,617	2,617	8	142 × 148	Acoustic Only	Real
SCTD	497	569	3	1109 × 596	Acoustic Only	Real
MAOUD	9,600	1,200	7	4096 × 3008	Optical, Acoustic	Simulated

The economic, labor and time costs of collecting data in a real underwater environment are high. Therefore, it is important to simulate and reproduce the real underwater environment in experimental waters. We have created a large-area underwater simulation scene using real sea sand, reefs, and high-precision coral models. To enhance scene complexity, we incorporated natural reef formations with rich geometric structures and undulating sandy terrain. The optical RGB images, laser point clouds, acoustic videos and images in this paper were collected in this underwater simulation scene. From the research point of view, our MAOUD dataset^14^ is important for underwater detection, enhancement and reconstruction tasks (Underwater biology detection and 3D scene reconstruction). From the application point of view, we have labeled seven categories of underwater targets and given them relevant category names, which are useful for data users in different domains and industries, for instance, training and evaluation in the field of underwater computer vision^15,16^, detection of biological populations in the aquaculture industry^17^, and underwater environments 3D reconstruction in the field of marine scientific research^18–21^.

Simultaneously, we use state-of-the-art image-laser-sonar underwater multimodal integrated detectors and ensure that the equipment is kinematically parameterized accordingly to ensure rigorous data collection. In addition, our integrated image-laser-sonar underwater multimodal detectors are pre-calibrated at the factory, which means that the data do not need to be manually calibrated. Our MAOUD dataset^14^ can contribute to the field of underwater environmental exploration, such as underwater detection, enhancement and 3D reconstruction based on acousto-optical data.

## Methods

### Collecting Data

**Fig. 1:**
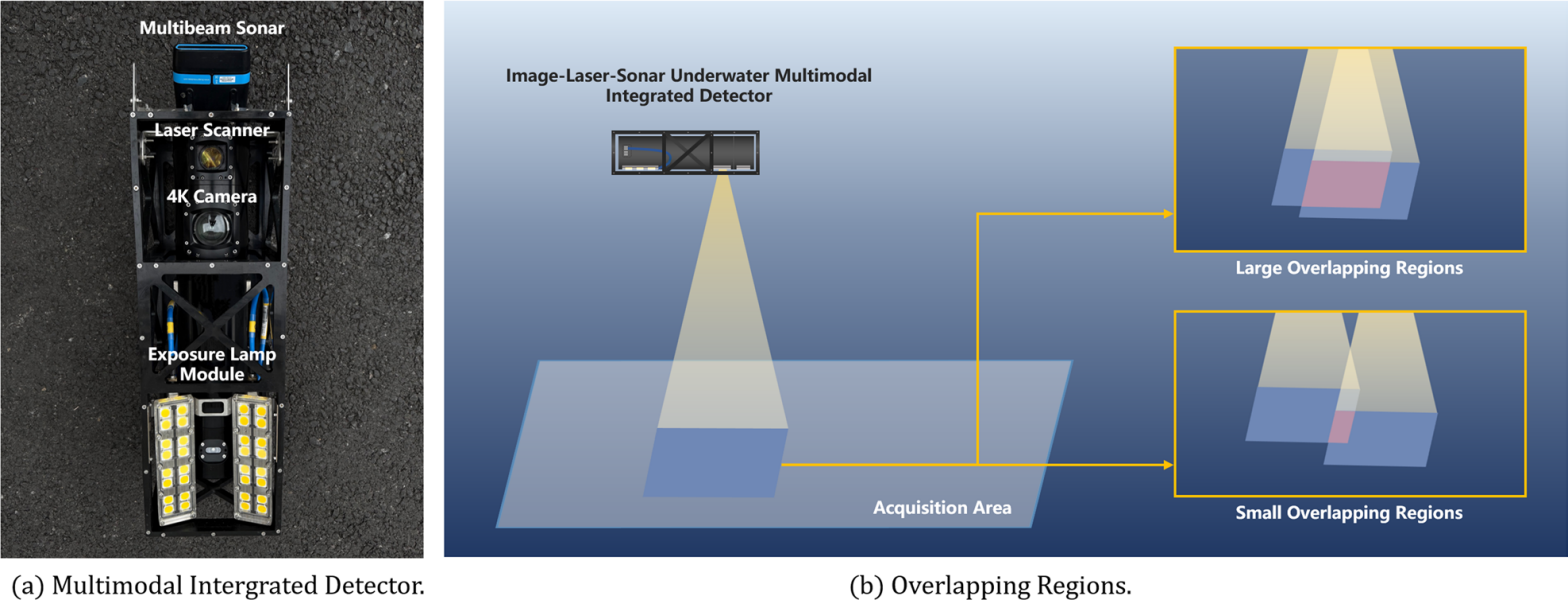
(**a**) Image-laser-sonar underwater multimodal integrated detector consists of four parts: a laser probe for reconstructing underwater scene point clouds, an optical 4K camera for capturing underwater RGB images, a multibeam sonar for capturing underwater acoustic videos and images, and an exposure lamp module for simulating different lighting conditions. (**b**) The image-laser-sonar underwater multimodal integrated detector reserves overlapping regions when acquiring image data in underwater simulation scene. We define areas with <30% overlapping area as small overlapping regions, and areas with >30% overlapping area as large overlapping regions.

We use the Image-Laser-Sonar underwater multimodal integrated detector based on Insight Micro - 1000m - Laser&12MP Stills with Oculus-M composition for acoustic-optical data acquisition, as shown in Fig. 1(a) The device consists of an optical 4K camera, a laser scanner, and a multibeam sonar, the optical HD probe is used to acquire underwater RGB images (resolution 4096 X 3008), the laser probe is used for point cloud reconstruction of the underwater scene, and the multibeam sonar is used to acquire underwater acoustic videos and images. The image-laser-sonar underwater multimodal integrated detector is suspended on a three-axis linear slider mechanism, which is used to control the detector to collect acoustic-optical data in different areas by changing the X and Y coordinates of the simulated underwater scene at a constant speed of 0.5 *m*/*s* and a constant water depth of 2 *m*.

**Fig. 2:**
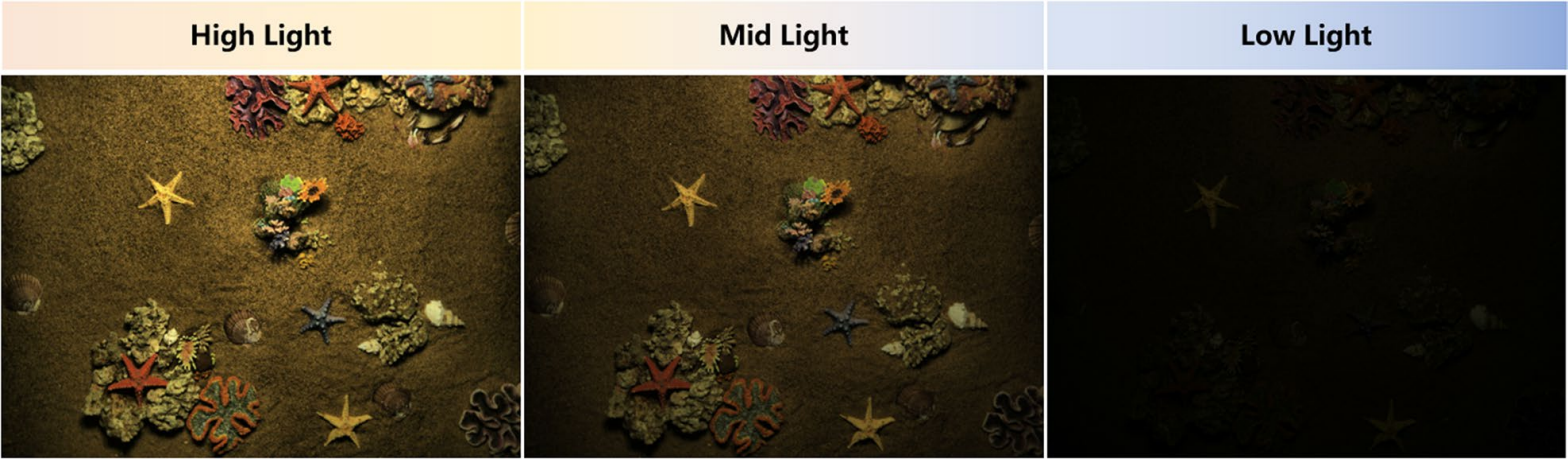
Data simulating different lighting conditions, where lighting is categorized as High, Mid, and Low, as determined by the exposure lamp module mounted on the body of the image-laser-sonar multimodal integrated detector.

**Fig. 3:**
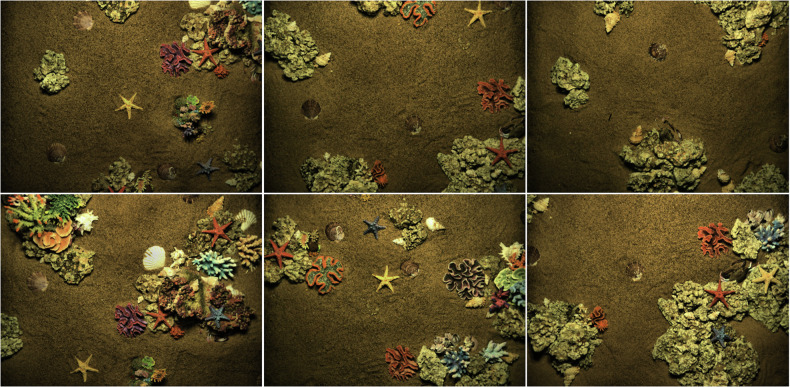
Arrangement of sea sand, reefs and target objects (scallops, starfish, conchs, crabs, corals, reefs, barnacles) in underwater simulation scenarios.

During the acquisition process, we ensure that the detector’s optical high-definition camera, laser scanner and multibeam sonar are always at a fixed height directly above the acoustic-optical data acquisition area in the underwater simulation scene, and strictly control the influence caused by the external light source, we use a dark room for data acquisition by cutting off the influence of natural light on the illumination fluctuation of the underwater simulation scene. We installed an exposure lamp module on the body of the image-laser detector to simulate three different underwater illumination levels, namely High light (illumination 2800 *lm*), Mid light (illumination 1600 *lm*) and Low light (illumination 200 *lm*), as shown in Fig. 2. At the same time, we employ artificially prepared simulated seawater (by proportionally adding compounds such as magnesium sulfate, sodium chloride, and potassium chloride) to replicate the spectral characteristics of real seawater, thereby avoiding the mismatch between freshwater and the actual marine environment caused by freshwater. We arranged seven categories of target objects in the simulated scene with the sea sand and rocks: scallop, starfish, conch, crab, coral, reef, and barnacle, as shown in Fig. 3. We chose the target object categories based on their frequency of occurrence in real marine ecosystems and their technical and commercial utilization value, for instance, training and evaluation in underwater computer vision^15,16^, exploration of biological populations in aquaculture and fisheries industries^17^, 3D exploration and reconstruction of underwater environments^18–20^.

After completing the construction of the underwater simulation scene, we manipulate the three-axis linear slider mechanism to hoist the image-laser-sonar underwater multimodal integrated detector to cruise and scan in the selected area to capture acoustic-optical data, and then adjust the area and record a large amount of data in turn. Meanwhile, in order to facilitate tasks such as image stitching and 3D preprocessing, we reserved enough overlapping regions for the acquisition area of neighboring routes to be used for feature point matching in such tasks, as shown in Fig. 1(b).

### Point Cloud Data

**Fig. 4:**
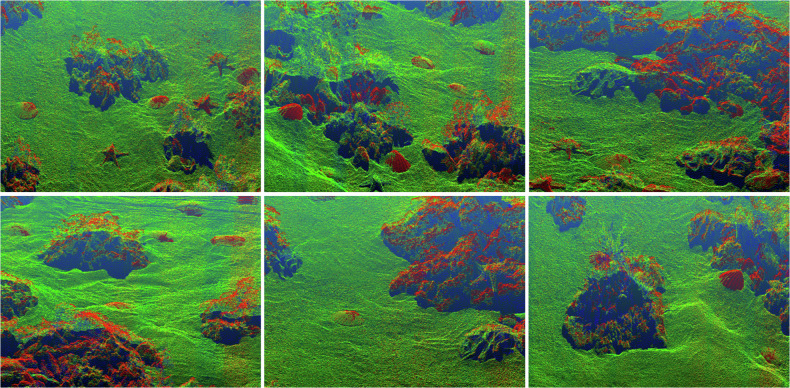
Example of a laser point cloud scan of the underwater scene, visualizing seabed topography, reefs and coral distribution.

We acquired the underwater images while also scanning the seabed with the help of an underwater laser detector and generated point cloud data as shown in Fig. 4. Each set of sequence images has a global point cloud corresponding to it. We achieve timestamp alignment of the point cloud with the images through factory pre-alignment and synchronized triggering of the image-laser-sonar underwater multimodal integrated detector to ensure cross-modal data consistency. Additionally, we utilize the Structure from Motion (SfM) algorithm to compute the sensor pose corresponding to each frame of imagery, which can be employed for tasks such as 3D reconstruction.

### Acoustic Video Data

**Fig. 5:**
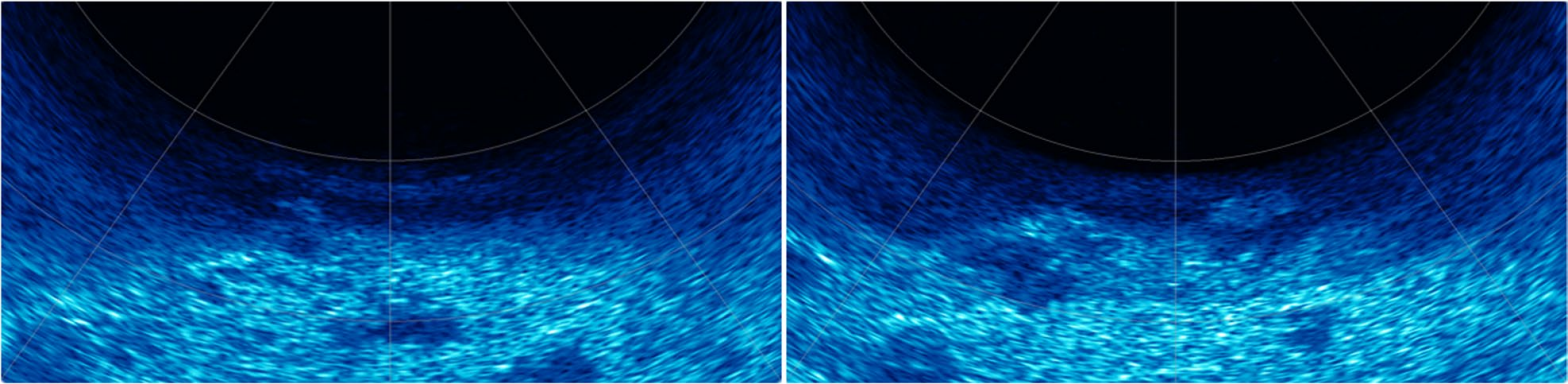
Example of keyframes from an acoustic video capture of the underwater scene where the target object is clearly visible.

We perform simultaneous acquisition of acoustic and optical data by an image-laser-sonar underwater multimodal integrated detector, in which the integrated multibeam sonar is used to acquire acoustic videos and images, as shown in Fig. 5. In order to suppress the noise interference generated by the device itself underwater, we use sound-absorbing materials to cover the underwater part of the device to ensure the accuracy of the data acquired by the multibeam sonar for the underwater simulation scene.

### Optic Object Annotation

**Fig. 6:**
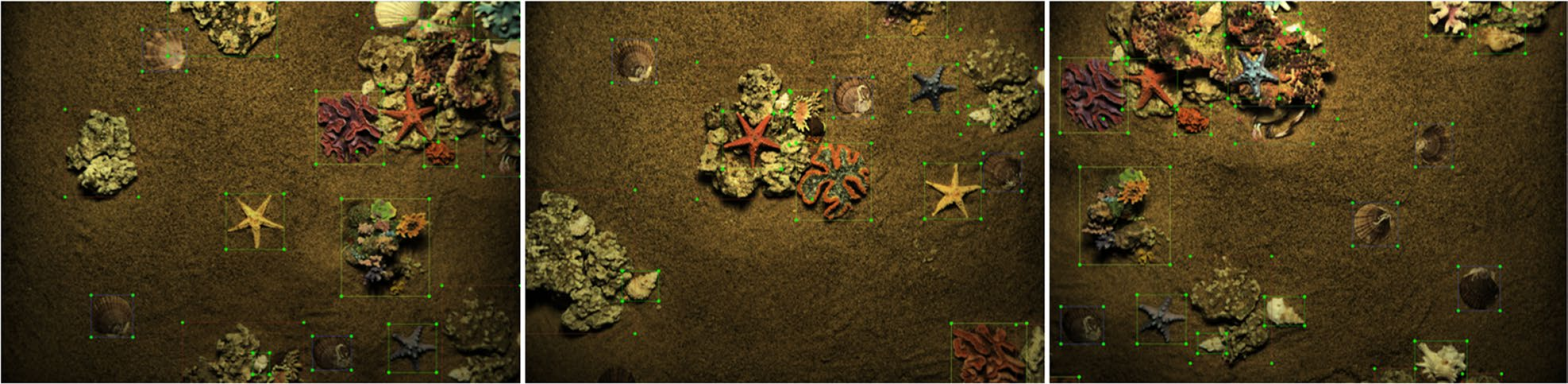
Example of underwater simulation scene example bounding box with category labels showing multiple specimens of 7 categories of target objects (scallops, starfish, conchs, crabs, corals, reefs, barnacles).

Considering the complexity of the underwater environment and the hidden nature of marine organisms, it is difficult for the labelers to judge the target category by experience and intuition alone when labeling. We set up two teams: one team uses annotation tool (Labelme) to initially annotate the dataset, creating polygons based on the target object’s shell edge punctuation so that the polygons fit the shape of the target object as much as possible, and displaying different colors for different target types; the other team checks and corrects omissions and mislabeling of the annotated dataset, and detects it with the annotated dataset. Among all the RGB images in the dataset, we only annotate the small overlapping region images (overlapping area <30%) to ensure that all the annotated images describe different regions of the target objects, and we do not annotate the large overlapping region images (overlapping area > 30%), but the original data of these images are still provided. As shown in Fig. 6, the category labels of the underwater simulation scene example mask show multiple specimens of seven target objects (scallop, starfish, conch, crab, coral, reef, barnacle), and the statistics on the number of these objects are shown in Fig. 8(a).

### Acoustic Object Annotation

**Fig. 7:**
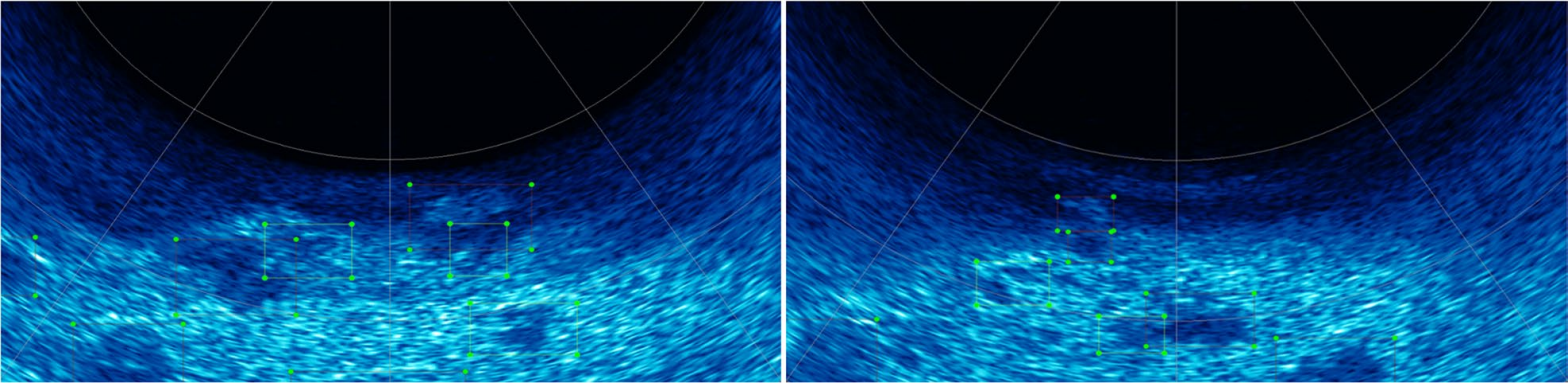
Example of an acoustic target bounding box for an underwater simulation scenario showing target objects such as reefs and corals with distinct acoustic identification features (reefs, corals).

Acoustic data collected by multibeam sonar in underwater simulated environments lacks rich texture information compared to optical images, which makes it difficult for the annotator to determine the target class based on experience and intuition alone during annotation. We address this problem by utilizing factory pre-collocation of multimodal data from the Image-Laser-Sonar underwater multimodal integrated detector. Our team set up bounding boxes based on target objects with distinct acoustic discrimination features in the acoustic images, as shown in Fig. 7, and confirmed the target categories against the optical images at the corresponding moments. Due to the resolution limitations of multibeam sonar, small invertebrate targets such as scallops and starfish cannot form echo signatures distinguishable from the seafloor background in acoustic imagery. Therefore, we only annotated large targets (reefs and corals) possessing actual visible acoustic identification features.

## Data Records

**Fig. 8:**
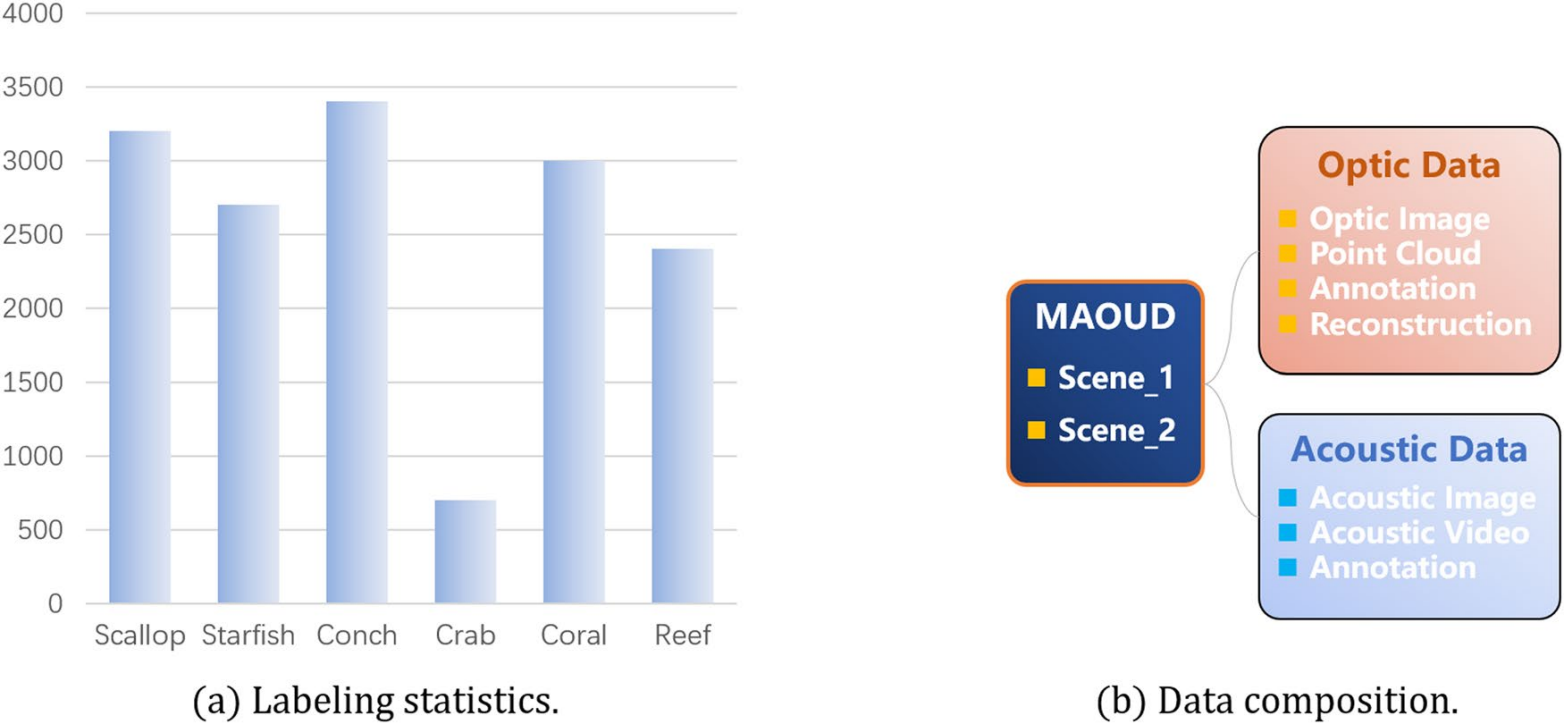
(**a**) Statistics on the number of labeled objects in underwater scenes. (**b**) Acousto-optical data composition and classification.

The dataset is available at Zenodo^14^. Our dataset comprises 7200 optical RGB images, 1200 labeled images, 24 sets of laser point clouds, 24 sets of acoustic videos, and 2400 acoustic images. The data is organized into 2 ZIP directories: “Scene_1.zip”, and “Scene_2.zip” The “Scene” folders comprise 7200 original RGB images, 1200 labeled images, and 24 sets of laser point clouds, 24 sets of acoustic videos, and 2400 acoustic images, organized into 2 distinct folders. The optical RGB images, the laser point clouds and the labeled images are located in the “Optic Data” folders, and the acoustic videos and images are located in the “Acoustic Data” folders, as shown in Fig. 8(b). The optical RGB images is further categorized into “High light,” “Mid light,” and “Low light” sub-folders based on llumination. Additionally, these sub-folders are subdivided into four groups according to the sequence of collection and the scanning area, labeled as “High light area_1”, “High light area_2”, “High light area_3”, and “High light area_4”. Simultaneously, we also upload metadata such as the sensor extrinsics and cross-modal transformation matrix alongside the dataset and archive it as “Meta.py” to support users in achieving spatial alignment of multimodal data.

## Technical Validation

### 2D Labeling Validation

We designed a three-level review mechanism to ensure the accuracy of optic and acoustic image annotation.

First, we use an image annotation tool (Labelme) to generate a bounding box based on the target contour, and establish corresponding color coding rules based on the preset seven types of targets (scallop, starfish, conch, crab, coral, reef, barnacle), which in turn generates the initial annotated image.

Second, we formed a review team consisting of marine biologists and computer vision researchers, assigned the initial labeled images to the team members, and manually reviewed them using cross-validation. In the cross-validation stage, we first checked the completeness of the annotation (e.g., whether there was any target omission), then checked the accuracy of the classification (e.g., morphological distinction between corals and reefs), and re-labeled if there was any controversy.

Third, we used the labeling consistency coefficient (Cohen’s Kappa) to quantify the degree of labeling consistency among different members, and the final overall Kappa value reached 0.93 (95% confidence interval 0.89-0.94), indicating that the labeling results are reliable.

In addition, for partially occluded target objects, we formulate the following rules to accomplish more accurate annotation: when the target occlusion area is less than 20%, they are annotated separately as independent individuals; when the target occlusion area is greater than 20%, the target boundaries are refined by morphological corrosion operations and follow the order of the occlusion to be annotated.

### Sensor Uncertainty Analysis

Our underwater acoustic-optical multimodal detector with built-in sonar, 4K camera and laser scanner is pre-calibrated at the factory, nsuring baseline accuracy of sensor data. Consequently, collected data requires no manual calibration. In terms of clarity impact metrics, a modulation transfer function (MTF) of 10 *lp*/*mm* was measured by a resolution test card. In terms of point cloud uncertainty metrics, a point cloud density of 120 points/*m*^2^ was achieved with a single-point positioning error of <3.5 *mm* and a relative error of <0.5% in the distance measurements at an overhead scanning distance of 2 *m*.

### Cross-Scenario Transferability Analysis

Our MAOUD dataset serves as a standardized training and validation benchmark for underwater acoustic-optical multimodal algorithms. The controllable nature of its simulated scenes eliminates interference from complex environments, facilitating algorithm performance verification. However, models trained on datasets collected from simulated scenes may encounter potential challenges when transferred to real underwater environments due to differences between simulated and real conditions:

1. Underwater simulated environments use fixed-spectrum spotlight illumination. Real underwater environments combine natural and ambient light, exhibiting complex spectral absorption effects that vary with depth;
2. Simulated seawater exhibits low turbidity and stable scattering, whereas real ocean environments feature varying degrees of turbidity influenced by ocean currents and suspended particles, resulting in dynamically changing scattering properties;
3. Underwater simulation environments possess lower background semantic diversity, whereas real seabed topography and backgrounds exhibit higher complexity.

These discrepancies may lead to issues such as insufficient adaptation under complex underwater optical conditions, poor robustness against dynamic water flow interference, and reduced target recognition accuracy in complex occlusion scenes within real ocean environments. To enhance the model’s cross-scenario transferability, we propose the following transfer optimization strategies for users:

1. Incorporate a medium-turbidity adaptive algorithm during model training to accommodate the optical characteristics of real underwater environments across varying turbidity levels;
2. Combine a small amount of real-world scene data with models trained on simulated data for joint training and fine-tuning, achieving domain adaptation from simulated to real data;
3. Establish feature parameters for different environments (light intensity, water scattering coefficient, terrain type) within the algorithm to enable scene adaptation.

### Limitations and Future Work

Despite the numerous advantages of our MAOUD dataset, its data are all collected in controlled experimental tank simulated scenes, which cannot fully replicate the complex optical properties, dynamic hydrodynamic disturbances and diverse seafloor topography of real ocean environments.

These differences and limitations will guide future work. Future datasets should consider expanding to include more diverse seafloor scenes. In terms of substrate, this could encompass seagrass beds, muddy seabeds, gravelly seabeds, and bedrock outcrop seabeds; Biologically diverse habitats could include biologically disturbed seabed, coral thicket, and macrobenthos aggregation zone; Artificial structures may include submarine pipelines, submarine cables, wreck debris, anchor marks and trawl marks.

## Usage Notes

In order to make it easier for users to download and run our dataset with the relevant algorithms, we archive each scenario as a separate zip file. Similarly, we have compressed all the code into a single zip file named “MAOUD_tools”. Users can process the original data using the scripts provided.

### Underwater Image Detection

**Fig. 9:**
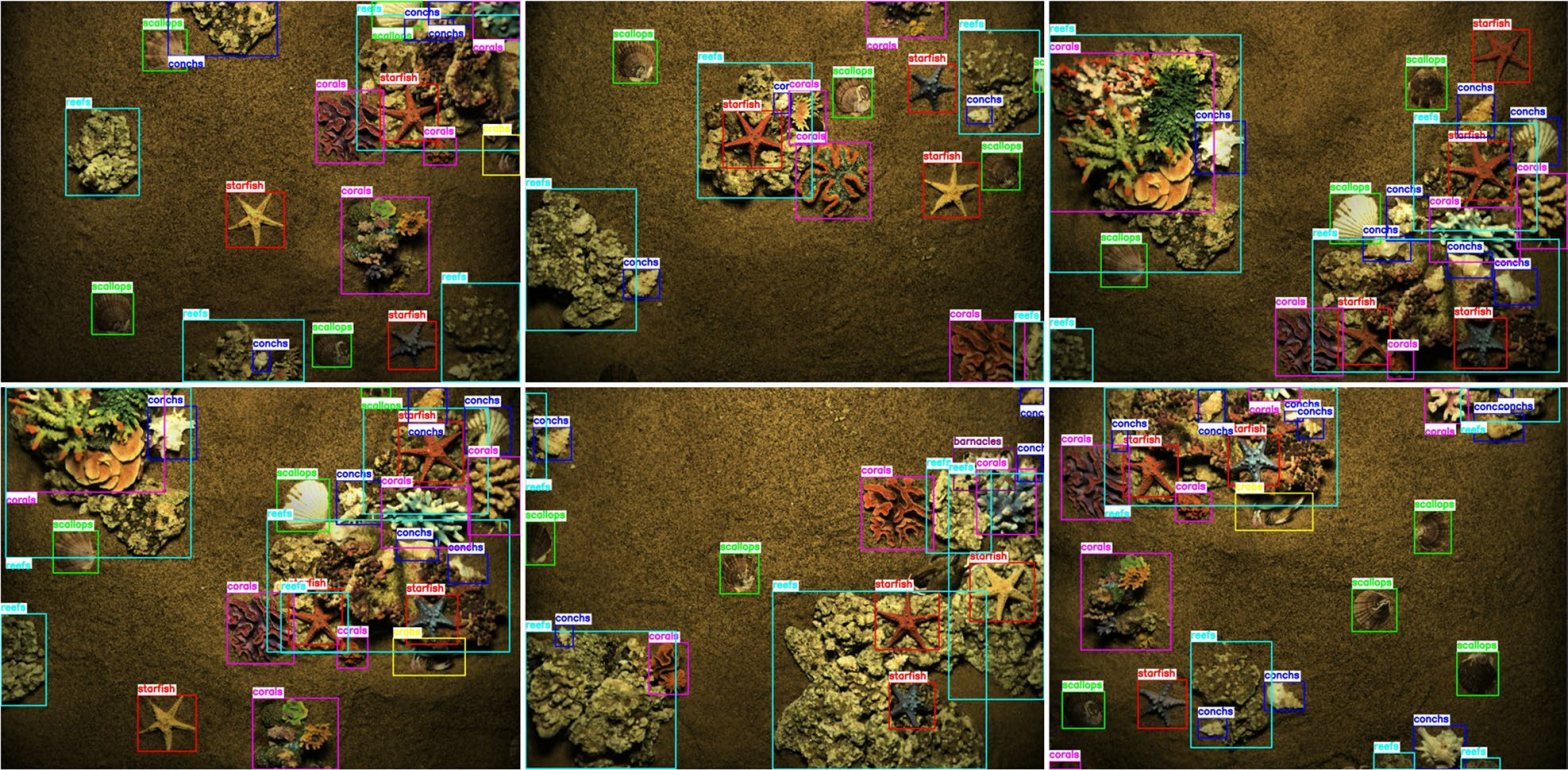
We tested the MAOUD dataset based on the YOLOv8 image detection algorithm, and the results show that this annotation method can ensure high accuracy and meet the research needs of underwater complex environments.

**Fig. 10:**
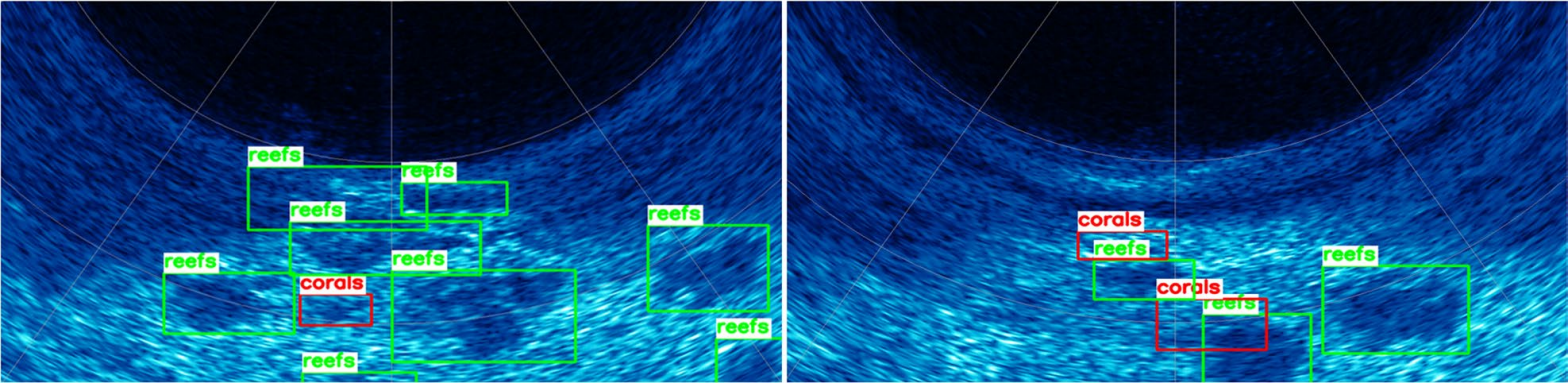
We tested the acoustic images based on the YOLOv8 image detection algorithm, and the test results show that the current annotation method ensures high detection accuracy for targets.

We evaluated the optic and acoustic data using the YOLOv8 algorithm^15^, as shown in Figs. 9 and 10. Underwater optical target detection *mAP@*0.5 = 92%. The test results demonstrate that this annotation method ensures high accuracy, meeting the research requirements for detecting optical images in complex underwater environments. Since we only annotated large targets with distinct acoustic identification features, our underwater acoustic target detection provides evaluation metrics exclusively for reefs *mAP@*0.5 = 91% and corals *mAP@*0.5 = 89%, validating the effectiveness of the acoustic modality for detecting large objects.

### Underwater 3D Reconstruction

We processed the original data using a 3D dataset preprocessing tool (Colmap), which includes camera parameter estimation, pixel-based feature point matching, and scene scale normalization. To ensure the usability of the 3D preprocessed data, we employed the 3D Gaussian Splatting^18^ for both visual and quantitative evaluation of the data. The quantitative evaluation metrics included:

1. Peak Signal to Noise Ratio (PSNR), measuring image similarity between synthetic and target views-higher values indicate lower distortion;
2. Structure Similarity Index Measure (SSIM), quantifying structural consistency between images-values closer to 1 indicate better preservation of texture and edge details;
3. Learned Perceptual Image Patch Similarity (LPIPS), which evaluates perceptual quality using a pre-trained neural network. Lower values indicate greater alignment between the synthesized view and human visual perception, which is crucial for assessing underwater scenes with significant variations in color and contrast.

**Fig. 11:**
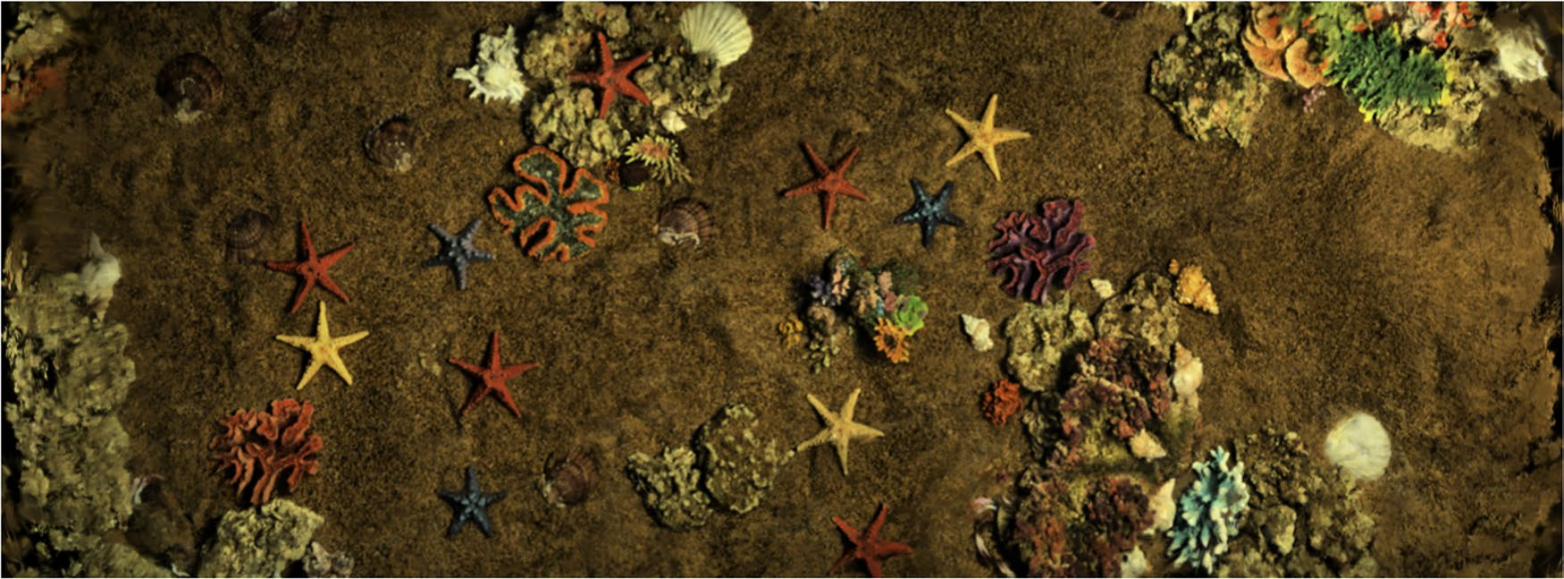
We tested the MAOUD dataset based on 3D Gaussian Splatting^18^. The result shows that our 3D preprocessing methodology ensures high quality underwater reconstruction and meets the research needs of underwater environments 3D reconstruction.

The visual results are shown in Fig. 11, and the quantitative results are presented in Table 4. The test results demonstrate that our MAOUD dataset and the preprocessing method ensure high reconstruction accuracy, thereby meeting the requirements for research and testing in 3D reconstruction within complex underwater environments.

**Table 4:** Quantitative evaluation metrics for 3D scene reconstruction based on our MAOUD dataset using 3D Gaussian Splatting^18^.

Scene	PSNR ↑	SSIM ↑	LPIPS ↓
S1H	36.249	0.977	0.051
S1M	34.593	0.954	0.089
S2H	36.235	0.971	0.053
S2M	34.414	0.949	0.091

“S1H” and “S1M” represent the high light and mid light environments of Scene 1, respectively. “S2H” and “S2M” represent the high light and mid light environments of Scene 2.

## Data Availability

The acoustic and optical data in our dataset is available from the Zenodo repository^14^ mentioned above.

## Code availability

We have released the Labelme and Colmap code along with the dataset in the Zenodo repository^14^, archived as “MAOUD_tools” and available online. In our labeling and preprocessing work, these tools work well under Ubuntu 20.04/22.04. “README.md” provides guidance for users along with the tools.
